# Two Atypical Cases of Nodular Gastritis: A Poorly Differentiated Gastric Adenocarcinoma and a Pseudo-Low Grade Gastric MALT Lymphoma

**DOI:** 10.4021/gr2010.02.170w

**Published:** 2010-01-20

**Authors:** Hye Jin Choi, Sun-Young Lee, Jung Hyun Lee, Dong Choon Seol, So Young Kim, Hae Jin Choi, Hyun Sik Park, Sung Noh Hong, Hye Seung Han

**Affiliations:** a Departments of Internal Medicine, Konkuk University School of Medicine, Seoul, Korea; b Departments of Pathology, Konkuk University School of Medicine, Seoul, Korea

**Keywords:** Nodular gastritis, *Helicobacter pylori*, Gastric cancer, Gastric mucosa associated lymphoid tumor

## Abstract

Nodular gastritis is a *Helicobacter pylori*-related gastritis with endoscopically proven gooseflesh skin-like nodularity in the gastric antrum. Although an association between nodular gastritis and gastric malignancies has been suggested, there is neither a treatment strategy nor a treatment guideline for this condition because of its relative rarity. We have recently experienced two cases of diffuse-type nodular gastritis invading both the antrum and corpus of the stomach with atypical findings that required specific treatments in two young females. The first patient was diagnosed with a suspicious low grade gastric mucosa associated lymphoid tissue (MALT) lymphoma lesion on a diffuse-type nodular gastritis, and was cured by *H. pylori* eradication. The second patient was diagnosed with a signet cell type gastric cancer on a diffuse-type nodular gastritis, and was cured by surgical resection. When considering the nature and significance of these gastric lesions, a link between nodular gastritis and gastric malignancy should be considered, especially in young women who have diffuse-type nodular gastritis involving both the antrum and corpus of the stomach.

## Introduction

Nodular gastritis is a gooseflesh or chicken skin appearing gastritis on the endoscopic finding. This special antral gastritis is characterized by an unusual military pattern with prominent lymphoid follicles in a biopsy specimen [1]. Although it is known as a common lymphofollicular proliferation of the gastric mucosa upon *Helicobacter pylori* (*H. pylori*) infection in children [2], it is relatively rare in adults. In East Asian countries where the prevalence of *H. pylori* infection is higher than the Western countries, nodular gastritis is reported up to 2.9% in adults [3]. Interestingly, female predominance is noticed in adults [3, 4], whereas no sex predilection in the presence of nodular gastritis has been reported in children.

*H. pylori* infection is known as a cause of several gastric diseases such as peptic ulcer disease, mucosa associated lymphoid tissue (MALT) lymphoma, and gastric cancer with accumulating evidences that chronic inflammation induce gene mutations, gene amplifications, and DNA dislocations in the affected cells [5]. Notably, a possible association between nodular gastritis and gastric cancer has been suggested with a characteristics of female predominance, dominant poorly differentiated or signet ring cell type gastric cancer, and a pangastritis leading to an advanced stage scirrhous cancer so called Borrmann type IV cancer that usually invades greater curvature side of the corpus [6]. However, it is difficult to know which nodular gastritis will regress spontaneously and which will progress to a gastric malignancy. Herein, we report two cases of nodular gastritis in young women with atypical findings that required specific treatment for their gastric lesions.

## Case Presentation

### Case 1

**Figure 1:**
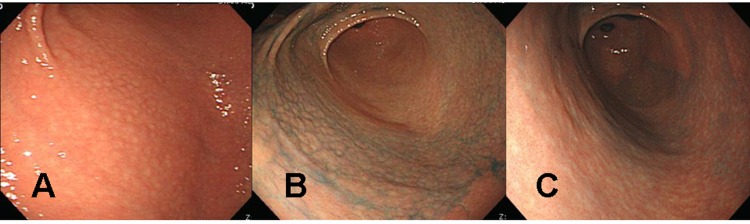
Endoscopic findings of the proximal antrum in the first patient (a 39 years old woman). (A) Initial findings showed micronodules measuring 2-5 mm in diameter with a smooth surface. The findings are observed from the antrum to the low corpus of the stomach, indicating a diffuse-type nodular gastritis. (B) There were no remarkable changes in nodular elevation on chromoendoscopic findings 1 month after H. pylori eradication, even after applying 0.2% indigo carmine spray. There was also no change in diffuse-type nodular gastritis since the previous examination. (C) Indigo carmine (0.2%) chromoendoscopy conducted 3 months after successful H. pylori eradication revealed, diminished nodular elevations.

**Figure 2:**
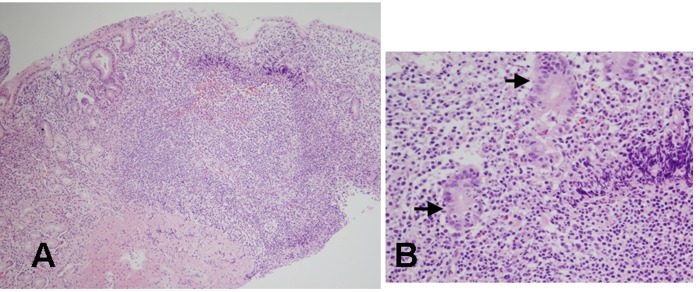
Histologic findings of biopsied specimens from the anterior aspect of the antrum in the first patient. (A) On microscopic findings at a low magnification, the nodular mucosa is expanded by a marked lymphoplasma cell infiltration with the presence of a prominent germinal center (H&E stain, x100). (B) On microscopic findings at a higher magnification, diffuse lymphoplasma cell infiltration is noted in the lamina propria, and a few lymphocytes are infiltrating the epithelium. Low grade gastric MALT lymphoma is suspected, but no definite lymphoepithelial lesion is found (H&E stain, x400).

A 39 years old Korean female visited our outpatient clinic because of indigestion. Five years ago, she was diagnosed as an iron deficiency anemia (IDA) and was taking iron supplement intermediately. She denied any past medical history or family history on gastrointestinal neoplasm. On arrival, her laboratory examination showed IDA with a serum hemoglobin level of 9.1 g/dL (normal range 12-16 g/dL), hematocrit 28.6% (normal range 36-48%), ferrum 22 µg/dL (normal range 65-157 µg/dL), total iron binding capacity 391 µg/dL (normal range 256-426 µg/dL), and ferritin 5.33 ng/mL (normal range 13-150 ng/mL). The fecal occult blood test showed negative finding. On the upper gastrointestinal endoscopic examination, diffuse mucosal nodularity with a cobblestone appearance was noticed (Fig. 1A). Biopsy was taken at the anterior aspect of the antrum, and was reported as *H. pylori*-infected lesion suggestive of low grade gastric MALT lymphoma (Fig. 2). Polymerase chain reaction study for IgH gene rearrangement was followed, and showed a negative finding.

*H. pylori* eradication was carried out, and follow up endoscopic examination was carried out after 4 weeks of therapy. Cobblestone appearance indicating diffuse-type nodular gastritis was still noticed from the antrum to lower corpus (Fig. 1B). Histopathological findings revealed neither a *H. pylori* infection nor a gastric MALT lymphoma in all the biopsied specimens from the antrum and corpus. Finally, the diagnosis was given as a nodular gastritis without an evidence of gastric MALT lymphoma-mimicking lesion after a successful *H. pylori* eradication.

After three months of regular iron intake, IDA was no longer detected. The blood tests showed serum hemoglobin level of 12.4 g/dL, hematocrit 36.0%, ferrum 174 µg/dL, total iron binding capacity 313 µg/dL, and ferritin 30.94 ng/mL. Follow up endoscopic examination with multiple biopsies showed an improvement of nodular gastritis (Fig. 1C). The patient is now being followed up with an annual endoscopic examination plan at our outpatient department.

### Case 2

**Figure 3:**
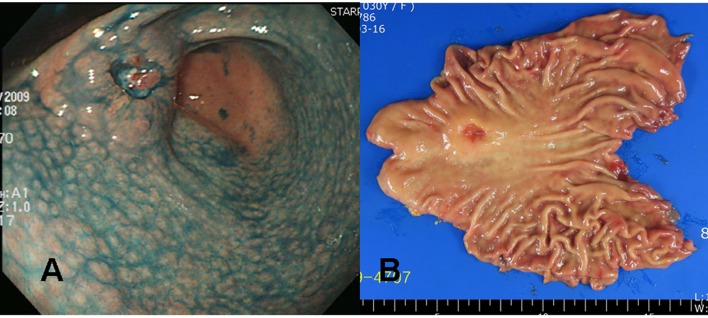
Gross findings of endoscopy and surgical specimens in the second patient (a 30 years old woman). (A) Chromoendoscopic findings with application of 0.2% indigo carmine showed diffuse mucosal nodularity evident on the antrum and lower corpus of the stomach, leading to a diagnosis of diffuse-type nodular gastritis. A round, elevated lesion with central ulceration can be seen on the anterior aspect of the proximal antrum. The edge and base of the ulcer are irregular and uneven, suggesting a cancerous change. (B) A 3.2 x 2.0 x 1.0 cm Borrmann type III gastric cancer was completely resected surgically by subtotal gastrectomy, with negative resection margins.

A 30 years old Korean female visited our health promotion center for a routine check-up. She had neither a remarkable past medical history nor a family history. She did not take any medication, although she felt intermittent upper epigastric discomfort recently. No abnormal finding was noticed on her initial blood test. On the upper gastrointestinal endoscopic examination, a 3 cm sized ulcerated lesion was detected (Fig. 3A). Biopsies taken at this lesion revealed poorly differentiated adenocarcinoma with a signet ring cell feature, and thus imaging studies were followed. Since there was no evidence of distant metastasis on the abdominal computed tomography, surgical resection was done.

**Figure 4:**
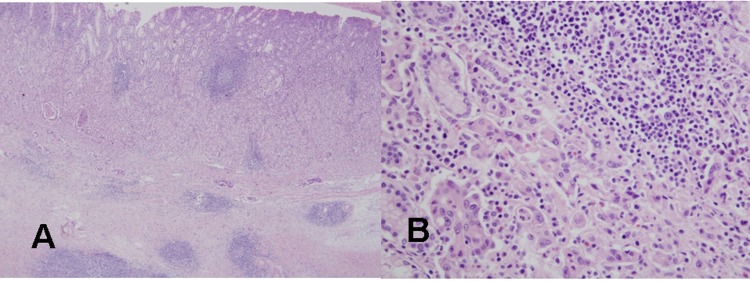
Histological findings of the resected gastric cancer in the second patient. (A) On microscopic findings at a low magnification, mononuclear cell infiltration with germinal centers is noted in the mucosa and submucosa around the gastric cancer (H&E stain, x40). (B) On microscopic findings at a higher magnification, a poorly differentiated tubular adenocarcinoma is evident near the lymphoid follicle in the mucosa (H&E stain, x400).

The tumor was resected by subtotal gastrectomy (Fig. 3B) with multiple lymph node dissections. Of 72 regional lymph nodes, 8 showed metastasis. Histopathological result showed a poorly differentiated adenocarcinoma invading down to the subserosal level with lymphatic and perineural invasions (Fig. 4). Venous invasion was not noticed on Hematoxylin and Eosin stain and factor VIII immunohistochemical stain. According to the Lauren classification, a diagnosis of mixed type indicating both intestinal-type and diffuse-type was given. Adjuvant chemotherapy was followed after the surgical resection. The patient is now on disease free status without any evidence of recurrence for 7 months.

## Discussion

**Figure 5:**
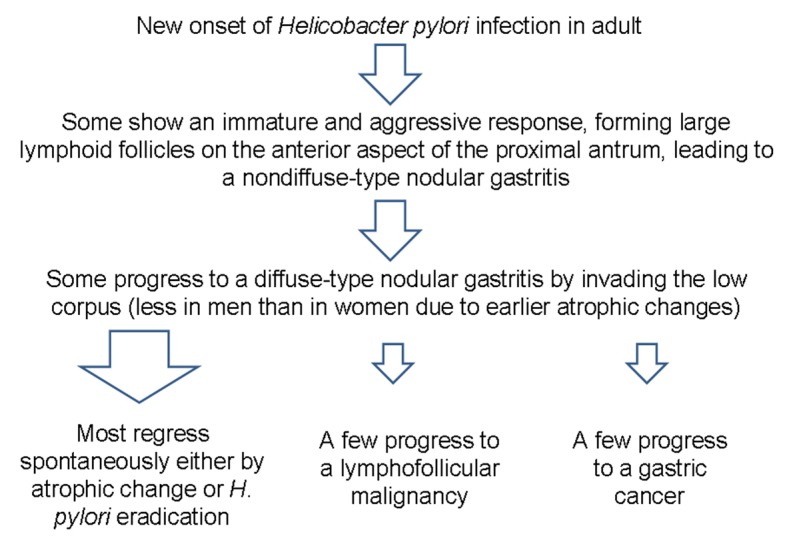
Schema of the progression of nodular gastritis to gastric neoplasms. In contrast to the case in children, novel H. pylori infection may lead to nodular gastritis in some adults, and some may progress to a diffuse-type nodular gastritis that invades both the antrum and body. A few cases of diffuse-type nodular gastritis may progress further to gastric neoplasm, especially in young women, due to fewer atrophic changes.

*H. pylori* infection can lead to several gastric malignancies [5, 6]. Although it is difficult to know which nodular gastritis will regress spontaneously or which will progress to a gastric malignancy, it seems that diffuse-type nodular gastritis invading both the antrum and corpus are prone to progress to gastric neoplasms (Fig. 5). It is known that B cell monoclonality precedes the development of MALT lymphoma in *H. pylori*-associated gastritis, and that lymphoproliferative pangastritis with a lymphoepithelial lesion would be a temporal change in *H. pylori*-induced B cell proliferation leading to a gastric MALT lymphoma [1, 7]. In addition, a possible association between the nodular gastritis and a poorly differentiated type gastric cancer with or without a signet ring cell feature has been suggested, especially in young women after puberty [6]. Because of such aggressive and invasive behavior of pangastritis leading to a Borrmann type IV cancer, surgery should be considered when there is no distant metastasis. Fortunately, both of our patients were cured owing to the early detection of their gastric lesions.

The cause of female predominance in nodular gastritis is uncertain, although there might be a gender specific immunity affecting the outcome of *H. pylori* infection leading to a severe form of nodular gastritis [3, 4]. Indeed, the prevalence of *H. pylori* infection is lower in women than in men before menopause [8]. However, it seems that once women get newly infected, it may lead to a more aggressive and immature response forming larger lymphoid follicles in the mucosal layer because of less atrophic change than men. As a result, higher prevalence of diffuse-type nodular gastritis involving both the antrum and corpus are more often noticed in women [4]. It is also known that antral diffuse nodularity disappear mainly from the lesser curvature of the antrum in association with the progress of mucosal atrophy [4]. This change probably starts earlier in men than in women, and thus leading to a lower prevalence of nodular gastritis in men. Taken as a whole, diffuse-type nodular gastritis is predominant in young women. Notably, our two patients were both women in their thirties, and were diagnosed as diffuse-type nodular gastritis.

Chemoprevention by *H. pylori* eradication therapy is usually indicated for gastric dysplastic lesions based on the Correa hypothesis [5]. In addition, gastric low grade MALT lymphoma is known to regress after *H. pylori* eradication [9]. However, optimal management of nodular gastritis in adults is unclear, despite previous reports showing some successful regressions of nodular gastritis after a successful *H. pylori* eradication [1, 10]. When considering the B cell clonality in gastric lymphoproliferative diseases, it is important to delineate the dynamic changes during the evolution of gastric MALT lymphoma from nodular gastritis [11]. Indeed, after a successful *H. pylori* eradication, number and size of the lymphoid follicles decrease significantly when compared to the groups without *H. pylori* eradication or failed eradication [12]. It seems that nodularity does not resolve immediately just after a successful *H. pylori* eradication, but resolves slowly taking more than three months to regress, as noticed in our first case. In summary, *H. pylori* eradication therapy may decrease the risk for MALT lymphoma in patients with nodular gastritis.

Herein, we reported two cases of diffuse-type nodular gastritis with significant gastric lesions in young women. Based on our cases, there might be a link between nodular gastritis and gastric malignancy especially in young women with diffuse-type nodular gastritis.
